# Size-Fractionated Filtration Combined with Molecular Methods Reveals the Size and Diversity of Picophytoplankton

**DOI:** 10.3390/biology10121280

**Published:** 2021-12-06

**Authors:** Xinze Shuwang, Jun Sun, Yuqiu Wei, Congcong Guo

**Affiliations:** 1 Institute of Marine Science and Technology, Shandong University, Qingdao 266237, China; shuwangxinze1996@163.com (X.S.); lonefeet@163.com (C.G.); 2 College of Marine Science and Technology, China University of Geosciences (Wuhan), Wuhan 430074, China; 3 State Key Laboratory of Biogeology and Environmental Geology, China University of Geosciences (Wuhan), Wuhan 430074, China; 4 Key Laboratory of Sustainable Development of Marine Fisheries, Ministry of Agriculture and Rural Affairs, Yellow Sea Fisheries Research Institute, Chinese Academy of Fishery Sciences, Qingdao 266071, China; weiyuqiu@163.com

**Keywords:** Western Pacific Ocean, picophytoplankton, high-throughput sequencing, size-fractionated filtration, flow cytometry

## Abstract

**Simple Summary:**

In order to accurately characterize the composition of the picophytoplankton community by size in the marine environment, we used a size-fractionated filtration plus a high-throughput sequencing molecular method. The results showed that this method can detect the composition of picophytoplankton in different particle size ranges in detail, and it is accurate in assessing the vertical distribution patterns and size of phytoplankton. This method can enrich the study of phytoplankton communities with diameters of less than 2 μm.

**Abstract:**

In this study, flow cytometry (FCM) and size-fractionated filtration, together with high-throughput molecular sequencing methods (SM), were used to investigate picophytoplankton. A particle separation filter and a higher-throughput sequencing method were used to evaluate the composition of a euphotic zone of picophytoplankton—especially picoeukaryotic phytoplankton—in the Western Pacific, and the results of flow cytometry, which is a classic way to detect picophytoplankton, were used as a standard to evaluate the reliability of the results of the SMs. Within a water column of 200 m, six water depths (5, 25, 50, 113 (DCM), 150, and 200 m) were established. In order to further study the particle size spectra of the picophytoplankton, size-fractionated filtration was used to separate water samples from each water depth into three particle size ranges: 0.2–0.6, 0.6–1.2, and 1.2–2 μm. A total of 36 (6 × 3 × 2) samples were obtained through PCR amplification of the 18S rRNA V4 hypervariable region and 16S rRNA, which were biased toward phytoplankton plastids, and then high-throughput sequencing was performed. The estimation of the picophytoplankton diameter relied on forward scattering (FSC) through FCM. The estimation of the vertical distribution and diameter of the picophytoplankton using the SM was consistent with the results with FCM; thus, we believe that the estimation of picophytoplankton composition with the SM has value as a reference, although the size-fractionated filtration seemed to cause some deviations. In addition to *Prochlorococcus* and *Synechococcus*, the SM was used to evaluate the composition of picoeukaryotic phytoplankton, which mainly included Prymnesiophycea (Haptophyta) (38.15%), Cryptophyceae (Cryptophyta) (22.36%), Dictyochophyceae (Chrysophyta) (12.22%), and Mamiellophyceae (Chlorophyta) (3.31%). In addition, the SM also detected Dinophyceae (Dinoflagellata) (11.69%) sequences and a small number of Bacillariophyceae (Diatom) (1.64%) sequences, which are generally considered to have large particle sizes. The results of the SM also showed that the picoeukaryotic phytoplankton were not evenly distributed in the euphotic layer, and the vertical distributions of the different picoeukaryotic phytoplankton were different. An analysis of correlations with environmental factors showed that temperature was the main environmental factor controlling the vertical distribution of picophytoplankton.

## 1. Introduction

Phytoplankton are important contributors to biogeochemical cycling in the world’s oceans and are a major provider of marine primary productivity, contributing about 50% of global primary productivity [1]. The structures of the diameters and compositions of phytoplankton communities affect the photosynthetic efficiency in primary production [2,3]. In subtropical oligotrophic waters, picophytoplankton (<2 μm) are the main primary producers [4,5,6]. Observations in the oligotrophic Pacific Ocean and Atlantic Ocean have revealed that picophytoplankton can account for approximately 60−80% of the total primary productivity [7,8]. In summary, picophytoplankton have been of great concern because of their significant impact on marine ecosystems and biogeochemical cycles. Picophytoplankton mainly include *Prochlorococcus*, *Synechococcus,* and picoeukaryotes [9,10].

The quantity and particle size of picophytoplankton are important data in evaluations of marine productivity and of their contribution to the global carbon cycle. The size of phytoplankton is fundamental in determining the fate of assimilated carbon [11]. It has been reported that the relative biomass contribution of picophytoplankton depends not only on abundance, but also on changes in cell size [12]. Conversely, studies have predicted that phytoplankton, such as diatoms [13,14], will shift to smaller phytoplankton species as the oceans warm [15,16,17], thus expanding areas with a higher cell abundance of picophytoplankton by the end of the 21st century, leading to unknown effects on the ecosystem [12]. However, in the past, picoeukaryotes were studied much less than *Prochlorococcus* and *Synechococcus* due to their relatively low abundance and high complexity and diversity [18,19]. Picoeukaryotes received little attention until recently [19,20,21,22], when it was confirmed that picoeukaryotes play an important role in the primary production of picophytoplankton [23]. Nevertheless, the composition of picoeukaryote communities in natural ecosystems remained poorly studied, especially in the open Western Pacific. Moreover, most studies actually found that the size of picophytoplankton extended to 3 μm, exceeding the standard of 2 μm, which may have biased the estimation of picoeukaryote composition [18,24,25,26,27].

**Figure 1:**
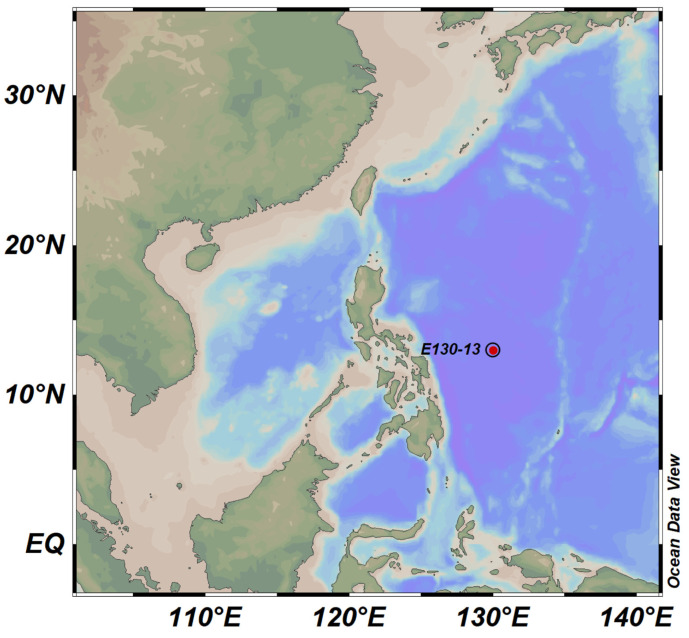
Sampling station (E 130°, N 13°).

Flow cytometry (FCM) has been widely used to study picophytoplankton abundance due to its ability to discriminate the three groups of picophytoplankton based on their amplitudes, shapes, and light signals [28,29]. Another method of obtaining picophytoplankton with different particle sizes is through size-fractionated filtration, which traps phytoplankton on membranes with different pore sizes and is considered a standard method for obtaining phytoplankton biomass samples [30,31,32]. In recent years, molecular techniques have become effective means of revealing the structures of phytoplankton communities because of their ability to detect small and rare species that are difficult to detect through traditional microscopic examinations and pigment analyses [33,34,35]. In this study, flow cytometry was used to quantify picophytoplankton and estimate their diameter in an oligotrophic euphotic zone of the Western Pacific Ocean (130° E, 13° N, Figure 1). In order to further clarify the structures of the composition and sizes of the communities of picophytoplankton, we used size-fractionated filtration to divide the picophytoplankton at each depth level into three particle size ranges—0.2–0.6, 0.6–1.2, and 1.2–2 μm—after which high-throughput sequencing was conducted. We named the combination of the two methods “SM” (size-fractionated filtration + molecular methods). The results of the FCM were compared as a standard with those obtained with the SM. The results showed that the SM was basically accurate in assessing the vertical distribution patterns and particle sizes of picophytoplankton, and it could measure the community composition of picophytoplankton with a diameter of ≤2 μm in a comprehensive way.

## 2. Materials and Methods

### 2.1. Sample Collection

This study was carried out on November 4, 2018 with the scientific RV “*Kexue*” in a 200 m water column in the Western Pacific Ocean (130° E, 13° N, Figure 1) with the support of the Open Cruise Project in the Western Pacific Ocean of the National Natural Science Foundation of China (NORC2018-09). A conductivity, temperature, and depth profiler (CTD) was used to collect water samples from water depths of 5, 25, 50, 113 (deep chlorophyll maximum—DCM), 150, and 200 m in a water column, and a sieve with a mesh size of 200 μm was used to remove larger plankton. Then, in each water depth, a total of 4 L of water was filtered with a setup of a size-fractionated filter holder on which were mounted a series of polycarbonate filter membranes (Millipore, Eschborn, Germany) with isopore sizes of 20, 2.0, 1.2, 0.6, and 0.2 μm (filters of with sizes of 20 and 2.0 μm were pre-filtered; only phytoplankton were analyzed on the filter membranes with pore sizes of 1.2, 0.6, and 0.2 μm in this study). In order to prevent cell breakage as a result of excessive pressure, we used a filtration pressure of ≤0.02 MPa. The filter membranes were placed in a 2 mL cryopreservation tube and immediately frozen in liquid nitrogen until the DNA was extracted. Then, 1.5 mL of seawater was collected from each water layer and fixed with paraformaldehyde (1% final concentration) for the flow cytometry analysis. To avoid loss of resolution and changes in cell size due to fixation or freezing, the FCM samples were left untreated at room temperature, left in the dark for 10–15 min, and then quickly frozen and stored in liquid nitrogen until the FCM analysis [36,37]. For the chlorophyll *a* sample, 2000 mL of seawater was serially filtered through a sieve with a mesh size of 20 μm, a 2 μm nylon membrane, and GF/F filters (diameter of 25 mm) under low vacuum pressure (<0.04 MPa); thereafter, they were stored in liquid nitrogen at –80 °C until processing.

Nutrient samples (200 mL) were collected in 500 mL PE vials and then immediately frozen at −20 °C for further analysis.

### 2.2. DNA Extraction and High-Throughput Sequencing

DNA was extracted from the samples using the DNeasy Power Water^®^ kit for water samples (Qiagen, Hilden, Germany) according to the instructions in the manual. The amount and quality of annotated DNA were observed with 1% agar gel electrophoresis, and the A260/A280 ratio was determined with a Nanodrop 2000 spectrophotometer (Thermo Fisher Scientific, Wilmington, DE, USA). Then, the hypervariable V4 of the 18S rRNA gene [38] primer and 16S rRNA gene primers, which are biased toward phytoplankton plastids [39], were used for amplification. For the amplification procedures, the protocols can be found elsewhere [28,29]. The PCR products were purified by using the MinElute^®^ PCR Kit (Qiagen, Hilden, Germany). The purified, amplified products were sequenced using an Illumina HiSeq2500 platform (Illumina, Inc., San Diego, CA, USA) via paired-end chemistry (PE250).

### 2.3. Flow-Cytometric Analysis of Picophytoplankton

**Figure 2:**
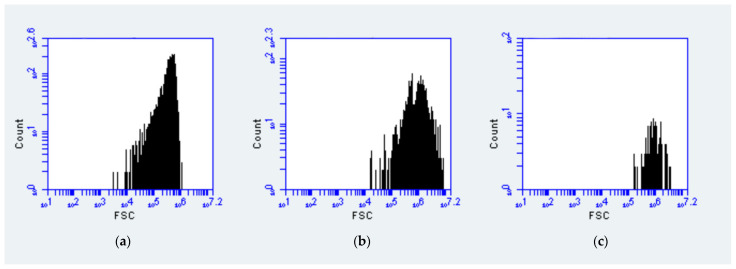
Forward-scatter frequency (FSC) of (**a**) *Prochlorococcus*, (**b**) *Synechococcus*, and (**c**) picoeukaryotic phytoplankton when counting cells.

According to different fluorescence signals and scatter characteristics, three picophytoplankton populations, namely, *Prochlorococcus, Synechococcus,* and picoeukaryotes, were classified and quantified by using flow cytometry (Becton–Dickinson Accuri C6) according to the standard methods detailed in the literature [29]. FCM was conducted with a laser emitting at 488 nm; the phycoerythrin and chlorophyll contained in *Synechococcus* could be excited with orange fluorescence (FL2) and red fluorescence (FL3) at 560–590 and 685 nm, respectively. *Prochlorococcus* and picoeukaryotes contain chlorophyll only, which can only emit red fluorescence; thus, *Synechococcus,* which can emit orange fluorescence, could be distinguished [40]. At the same time, forward-scattering light (FSC) was used to characterize the cell size in order to distinguish *Prochlorococcus* and picoeukaryotes. *Prochlorococcus* showed weak fluorescence due to their small cells, while picoeukaryotes showed strong fluorescence due to their large cells [41]. Histograms of examples of FSC frequencies of the three picophytoplankton groups are shown in Figure 2. According to the different FSC characteristics of the cells, the cell diameter was found by using FCM. We assumed that the optical signals of the mean cell diameters of *Prochlorococcus* (0.6 μm) were similar to those of the normalized beads. This hypothesis was similar to the pattern found by Blanchot et al. [42]. The cell sizes were estimated with respect to the biovolumes on the basis of the mean FSC frequencies of *Prochlorococcus*, *Synechococcus*, and picoeukaryotic phytoplankton, and they were measured relative to the mean cell diameters, with reference to Wei et al. [41,43]. The empirical relationship between the mean FSC (MFSC) and cell diameter (dcell) shown was the following: dcell = dbead_(*Prochlorococcus*)_ ^(MFSCcell/MFSC*Prochlorococcus*)^ [41].

### 2.4. Environmental Parameter Measurement

The nutrient concentrations of nitrate (NO_3_^−^-N), nitrite (NO_2_^−^-N), ammonium (NH_4_^+^-N), silicate (SiO_3_^2−^-Si), and phosphate (PO_4_^3−^P) were measured using a Technicon AA3 AutoAnalyzer (Bran+Luebbe, Norder-stedt, Germany). Dissolved inorganic nitrogen (DIN; the sum of NH_4_^+^, NO_3_^−^, and NO_2_^−^) was analyzed by using the method of copper–cadmium column reduction [44]. Dissolved inorganic phosphorus (DIP; PO_4_^3−^P) and silicate (DSi; SiO_3_^2−^-Si) were analyzed by using molybdenum blue reagents and standard molybdic acids, respectively [45,46]. The detection limits of our AA3 AutoAnalyzer for NH_4_^+^-N, PO_4_^3−^-P, NO_3_^−^-N, NO_2_^−^-N, and SiO_3_^2−^-Si were 0.040, 0.024, 0.015, 0.003, and 0.010 μmol L^−1^, respectively. For the undetectable nutrients, we imposed a minimum nutrient concentration of 0.01 µmol L^−1^ to avoid issues with the detection limits.

For the analysis of chlorophyll *a*, extraction was carried out in 5 mL of 90% acetone (4 ℃ for 24 h). After the removal of the filter membranes, the Chl *a* concentrations were assessed on a CE Turner Designs Fluorometer according to the acidification method of Welschmeyer [47].

### 2.5. Data Analysis

For molecular data, the original sequences were filtered using the QIIME v1.8 (Quantitative Insights into Microbial Ecology) [48] software, and high-quality sequences were obtained by splicing, de-priming, de-joining, and excluding sequences with low-quality scores. The Flash v1.2.7 software [49] was used for sequence splicing. The tags of paired-end reads were spliced by overlapping the relationships between reads. USearch v7.0.1090 [50] was used to cluster the spliced tags at 97% similarity. Then, Uchime v4.2.40 [51] was used to remove the chimeras generated by the PCR amplification from the representative OTU sequence according to the Denovo method, and the representative OTU sequence was obtained. Species annotation was carried out on the representative OTU sequence with the RDP Classifer (V2.2) software, and the databases used for the comparison were SILVA and Greengene. The OTU without annotation results was removed, and the confidence threshold was set to 0.6. Using the species annotations and OTU readings, we obtained the distribution of the OTU abundance for all samples. OTUs with an abundance of less than 0.005% of the total dataset may have affected the estimations of the abundance and diversity and were excluded in subsequent analyses. The OTUs that were annotated as archaea, embryogenic plants, fungi, zooplankton, or other sequences were discarded in this study. MEGA X [52] was used to construct a phylogenetic tree together with a sequencing sequence, and resampling was performed 1000 times with the bootstrap method to verify the stability of the cluster. Further, this phylogenetic tree was edited with the online webpage Evolview [53]. The data presented in this study are available from NCBI under PRJNA748570 and PRJNA747866.

The vertical abundance of the phytoplankton was plotted by using the Origin software (v2018). The R software was used for the diversity analysis and Pearson correlation analysis.

## 3. Results

### 3.1. Environmental Parameters

**Figure 3:**
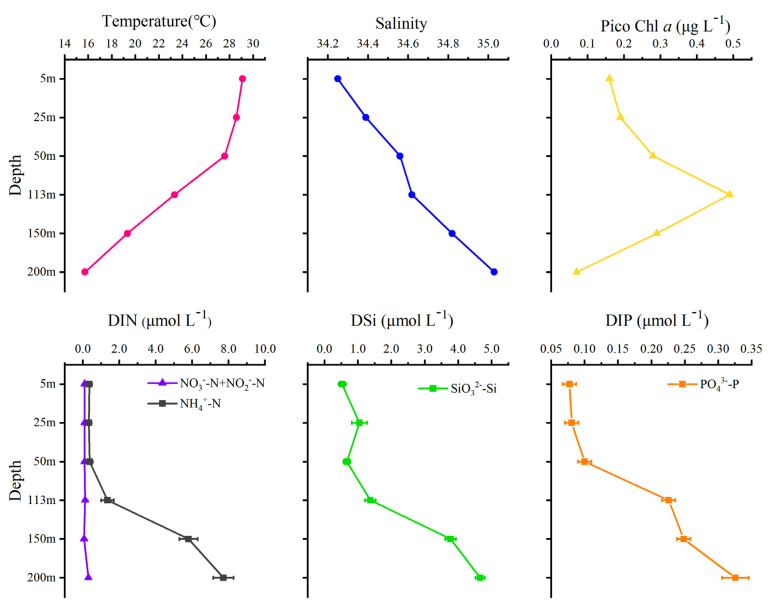
Environmental parameters along the vertical profile.

The temperature along the profile was between 15.7 and 29.1 ℃, with a thermocline appearing at 50 m. The salinity varied from 34.2 to 35.0 and increased with increasing depth, but the variation was not obvious. The concentrations of nutrients (DIN, DSI, and DIP) increased with depth. The highest concentration of DIN was 8.02 μmol L^−1^ (200 m), the lowest concentration was 0.42 μmol L^−1^ (5 m), and the average was 2.8 ± 3.02 μmol L^−1^. The highest concentration of DSI was 4.65 μmol L^−1^, the lowest was 0.53 μmol L^−1^, and the average was 2.01 ± 1.60 μmol L^−1^. The DIP concentration was relatively low, with a maximum of 0.33 μmol L^−1^ and a minimum of 0.08 μmol L^−1^; the average was 0.18 ± 0.1 μmol L^−1^. According to the changes in temperature and nutrients, the water column had a shallow mixing layer from 50 to 113 m, and an obvious stratification appeared. The maximum value of the pico chlorophyll *a* appeared at 113 m (0.49 μg L^−1^, Figure 3).

### 3.2. Vertical Distribution Patterns and Estimation of the Size of Picophytoplankton Based on Flow Cytometry

**Figure 4:**
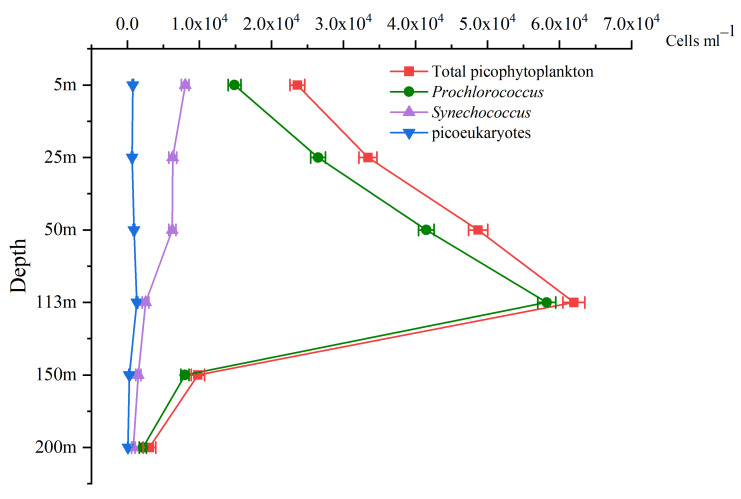
Flow cytometry results for the three picophytoplankton groups.

The results of flow cytometry showed that *Prochlorococcus* was dominant in the water column, and the vertical distribution pattern of the picophytoplankton was determined. The total picophytoplankton abundance was 3.01 ± 2.07 × 10^4^ cells mL^−1^; that of *Prochlorococcus* was 2.52 ± 1.95 × 10^4^ cells mL^−1^, while those of *Synechococcus* and picoeukaryotes were 4.24 ± 2.72 × 10^3^ cells mL^−1^ and 6.60 ± 4.06 × 10^2^ cells, respectively. The same vertical distribution patterns were observed for *Prochlorococcus* and the total picophytoplankton; the cell abundance first increased and then decreased with depth, reaching its maximum (6.20 ± 0.15 × 10^4^ cells mL^−1^ for total picophytoplankton, 5.82 ± 0.13 × 10^4^ cells mL^−1^ for *Prochlorococcus*) at a depth of 113 m and its minimum (3.05 ± 0.87 × 10^3^ cells mL^−1^ for total picophytoplankton, 2.14 ± 0.51 × 10^3^ cells mL^−1^ for *Prochlorococcus*) at a depth of 200 m. While the cell abundance of *Synechococcus* gradually decreased with the increase in depth, the maximum value (8.03 × 10^3^ ± 0.54 cells mL^−1^) appeared in the surface layer. The cell abundance of the picoeukaryote cells first increased and then decreased with increasing depth, but the maximum value (1.31 × 10^3^ ± 0.12 cells mL^−1^) was at 113 m (DCM) (Figure 4).

The results from the estimated mean FSC method indicated that the mean cell diameter of *Prochlorococcus* (0.60 ± 0.22 μm) was the smallest, followed by those of *Synechococcus* (0.83 ± 0.10 μm) and picoeukaryotic phytoplankton (1.20 ± 0.47 μm).

### 3.3. Vertical Distribution Patterns and Particle Size Composition of Picophytoplankton Based on High-Throughput Sequencing

One prokaryotic picophytoplankton phylum (Cyanobacteria) and six eukaryotic picophytoplankton phyla (Haptophyta, Cryptophyta, Chrysophyta, Chlorophyta, Dinoflagellata, Ochrophyta) were detected through the high-throughput sequencing of the SM samples.

**Figure 5:**
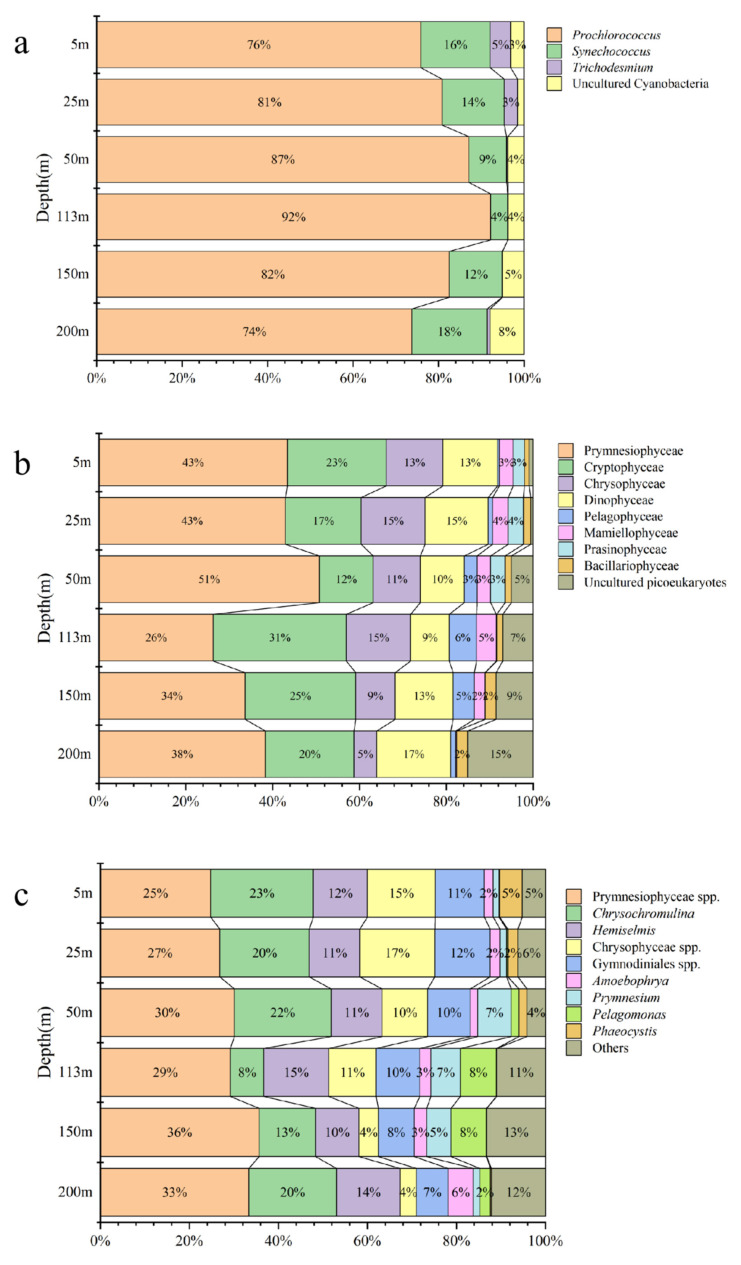
Relative abundance of picophytoplankton in the vertical direction based on high-throughput sequencing. Proportions of <2% are not marked. (**a**) the relative abundance of the top 10 picoeukaryotes; (**b**) the relative abundance of the top 10 picoeukaryotes at the class level; (**c**) the relative abundance of the top 10 picoeukaryotes at the genus level.

For the prokaryotic picophytoplankton, in addition to *Prochlorococcus* and *Synechococcus,* high-throughput sequencing of the SM samples also revealed the presence of *Trichodesmium* (Figure 5a). Concordantly with the flow cytometry results, the high-throughput sequencing results also showed that *Prochlorococcus* (84.28%) and *Synechococcus* (10.70%) dominated the picoprokaryotic phytoplankton in the water column, and *Trichodesmium* (1.76%) only appeared in the surface layer. The results of the SM showed a greater diversity of picoeukaryotes (Figure 5b,c). At the class level, Prymnesiophycea (Haptophyta) (38.15%), Cryptophyceae (Cryptophyta) (22.36%), Dictyochophyceae (Chrysophyta) (12.22%), Dinophyceae (Dinoflagellata) (11.69%), Mamiellophyceae (Chlorophyta) (3.31%), and Prasinophyceae spp. (Chlorophyta) (1.58%) were the main picoeukaryotic phytoplankton in the water column. A small number of Bacillariophyceae (Diatom) (1.64%) sequences were also detected. At the genus level, Prymnesiophyceae spp. (Haptophyta) (29.56%), *Chrysochromulina* (Haptophyta) (16.42%), *Hemiselmis* (Cryptophyceae) (12.40%), Gymnodiniales spp. (Dinophyceae) (9.88%), *Amoebophrya* (Dinophyceae) (2.47%), *Prymnesium* (Haptophyta) (4.77%), *Pelagomonas* (Chrysophyta) (3.92%), and *Phaeocystis* (Haptophyta) (1.52%) were the main picoeukaryote genera in the water column.

**Figure 6:**
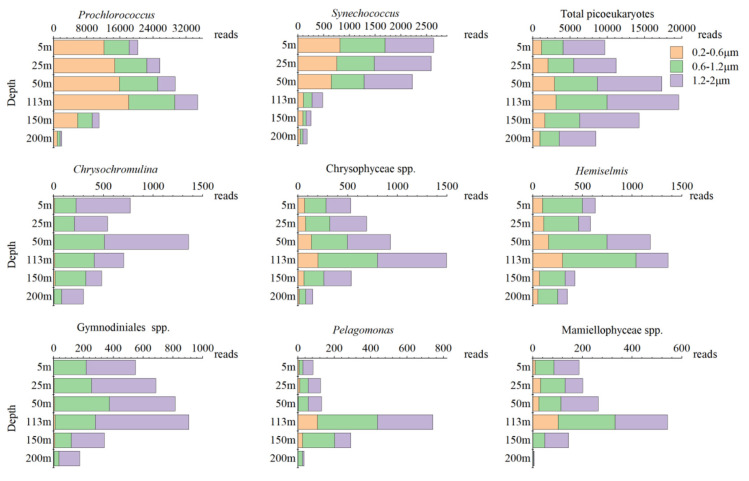
Vertical variations in the numbers of reads and variations in the three particle size ranges in the three picophytoplankton groups and six dominant picoeukaryote genera. Notice the values on the horizontal axis.

Figure 6 shows the changes in the numbers of reads of the genera with the highest abundances in each class in the vertical direction and within the three particle size ranges. The variations in the numbers of reads in the vertical profile of each genus roughly represent their abundance. The vertical changes in the numbers of reads of *Prochlorococcus*, *Synechococcus,* and total picoeukaryotes were consistent with the results of flow cytometry. The number of *Prochlorococcus* reads first increased and then decreased with the increase in depth, with a shallow peak at 113 m and a sharp decrease at 150 m. The number of *Synechococcus* reads decreased gradually with the increase in depth, while the number of total picoeukaryote reads first increased and then decreased with the increase in depth, and it reached its maximum at 113 m (Figure 6). The different picoeukaryotes had different distribution patterns in the vertical direction. In general, picoeukaryotic phytoplankton tended to accumulate in the DCM. *Chrysochromulina* had a large number of reads at the surface and in the thermocline (50 m). The vertical distribution of Chrysophyceae spp. was similar to that of *Hemiselmis*, and the number of reads increased and decreased with increasing depth, reaching the highest level in the DCM. Gymnodiniales spp. was the main pico-dinoflagellate detected with the SM method, and its sequence existed in the whole euphotic zone. The number of reads of Gymnodiniales spp. gradually increased from 5 to 113 m and reached its maximum at 113 m, then had a distinct decrease in the deep euphotic zone. *Pelagomonas* had a larger number of reads at 113, 150, and 200 m, and the highest number of reads was found at 113 m, though its read number was extremely low from the surface to 50 m deep. The read number of Mamiellophyceae spp. was generally higher from the surface to 113 m, and it reached its highest at 113 m, while the number of reads decreased sharply below 113 m, and the abundance was low at 200 m (Figure 6).

It was obvious that the relative abundances of the three picophytoplankton groups were different in the three particle size ranges, and the particle size composition structures were also different at different depths (Figure 6, Table 1). For *Prochlorococcus*, most sequences in the whole water column were distributed in the particle size range of 0.2–0.6 μm (59.86%), with the smallest proportion in the particle size range of 1.2–2 μm (12.21%). The *Synechococcus* sequences had a similar distribution in the particle size ranges of 0.2–0.6μm (31.46%) and 0.6–1.2 μm (29.32%), with a relatively large proportion in the particle size range of 1.2–2 μm (39.22%). For picoeukaryotes, most sequences were distributed in the particle size range of 1.2–2 μm (52.88%), and the number of sequences was the lowest in the particle size range of 0.2–0.6 μm (14.71%) (Table 1). However, the vertical particle size compositions of these three picophytoplankton groups were different. For instance, with the increase in depth, the relative abundance of *Prochlorococcus* gradually changed from being higher in the range of 0.2–0.6 μm to being higher in the ranges of 0.6–1.2 and 1.2–2 μm. However, the particle sizes of *Synechococcus* and total picoeukaryotes did not change much in the vertical direction (Figure 5). Most of the genera of picoeukaryotes had their largest numbers of reads in the size range of 1.2–2 μm, but there were also many reads in the size range of 0.2–0.6 μm in some picoeukaryotes, such as Chrysophyceae spp. and Mamiellophyceae spp., of which several genera have been shown to belong to picophytoplankton [54,55,56,57]. However, reads of *Hemiselmis* in the size range of 0.2–0.6 μm may have been errors caused by the separation and filtration according to particle size because its cells are fragile.

### 3.4. Diversity and Phylogenetic Relationships of Picophytoplankton

**Figure 7:**
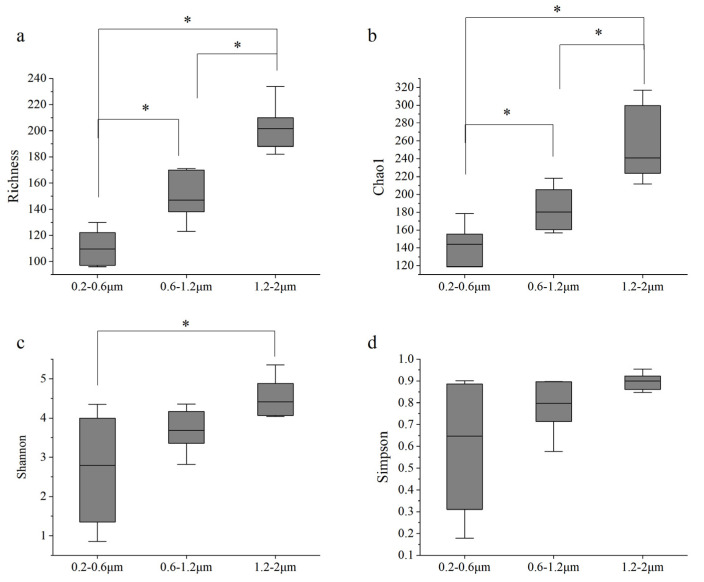
Differences in the diversity of picophytoplankton with different particle sizes. (**a**) Richness (ANOVA); (**b**) Chao1 (ANOVA); (**c**) Shannon (ANOVA); (**d**) Simpson (Kruskal–Wallis). Significance marker *: *p* < 0.05.

There were no significant differences in picophytoplankton diversity in the vertical direction (*p* > 0.05). Nevertheless, the diversity index of picophytoplankton in the different diameter ranges showed significant differences. With the increase in diameter, the picophytoplankton diversity index gradually increased. The richness index and Shannon–Wiener (H’) diversity index of picophytoplankton with diameters of 1.2–2 μm were significantly higher than those of picophytoplankton with diameters of 0.2–0.6 and 0.6–1.2 μm (*p* < 0.005, Figure 7). The reason was that most picophytoplankton were between 1.2 and 2 μm in size, including *Prochlorococcus*, *Synechococcus,* and most picoeukaryotes.

**Figure 8:**
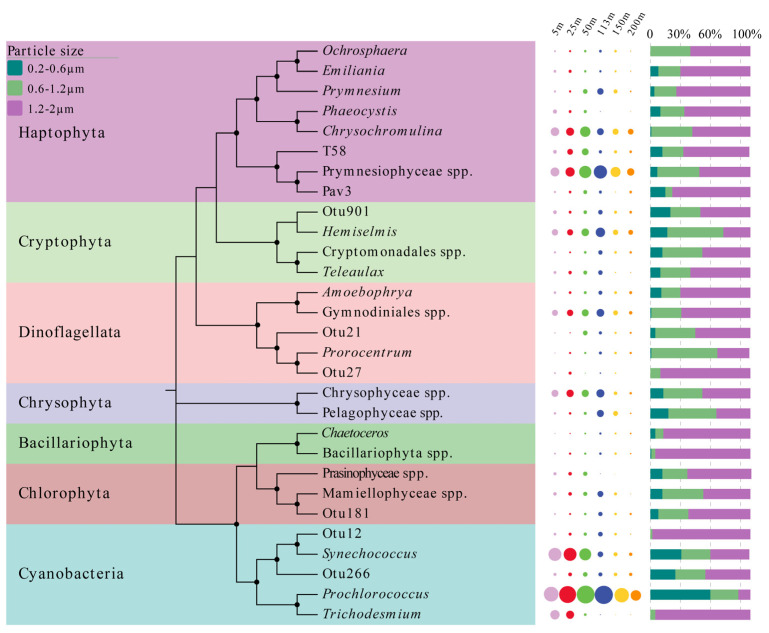
The phylogenetic evolutionary tree of picophytoplankton. The topology of the tree was inferred by bootstrap resampling 1000 times, and bootstrap values greater than 50% were labeled with black dots at the branches. The size of the point represents the abundance of picophytoplankton in the corresponding depth, and the larger the point is, the greater the number of sequences is. The bar chart represents the abundance of picophytoplankton in the corresponding diameters, and the longer the bar is, the higher the abundance is.

A maximum-likelihood phylogenetic tree was established to assess the phylogenetic relationships among the picophytoplankton, with the size of the dots behind the tree indicating the abundance of phytoplankton in each depth; the bar chart shows the distribution of the relative abundance of picophytoplankton in the diameter ranges of 0.2–0.6, 0.6–1.2, and 1.2–2 μm (Figure 8). Among the picophytoplankton, the genera of Haptophyta were the most frequently found, followed by those of Cryptophyta and Dinoflagellates. The dot plot shows that Haptophyta, Cryptophyta, Cyanobacteria (*Prochlorococcus* and *Synechococcus*), Chlorophyta, Chrysophyta, and some genera of Dinophyceae (Dinoflagellates) were relatively abundant picophytoplankton groups in the column. Some unclassified OTUs were closely related to the genera of Haptophyta, Cryptophyta, Dinophyceae, and *Synechococcus*, respectively, suggesting that the abundance of these picophytoplankton may have been underestimated. The OTUs of some nano-phytoplankton, such as *Ochrosphaera* [58], *Emiliania* [59], and Bacillariophyta, were also recovered, although their abundance in the water column was low.

### 3.5. Comparison of the FCM and SM Results

We compared the results of FCM with those of the SM in terms of the vertical distribution, particle size, and correlation with environmental factors. In terms of the vertical distribution, the results of FCM and SM were consistent: The abundance of *Prochlorococcus* and picoeukaryotes first increased and then decreased with the increase in depth, reaching the maximum at 113 m (DCM), while the abundance of *Synechococcus* decreased with the increase in depth (Figure 4 and Figure 6). This indicates that the SM is reliable in evaluating the vertical distribution of picophytoplankton.

To quantify the particle sizes of the three picophytoplankton obtained with the SM, we took the average values of each particle size range and multiplied them by the corresponding percentages to obtain approximate particle sizes. The formula was as follows: Average (Range_max_ + Rang_min_) × N%, where Range_max_ and Range_min_ were the upper limit and lower limit of the particle size range, respectively, and N% was the proportion of picophytoplankton in the particle size range. Then, we obtained the approximate particle sizes of the three picophytoplankton: *Prochlorococcus* was about 0.69 μm, *Synechococcus* was about 1.02 μm, and picoeukaryotes were about 1.21 μm. The results of the estimation with the SM all fell within the ranges of the results of the estimation with FCM (Table 2), which indicates that the SM was accurate in estimating the particle sizes of the three types of picophytoplankton.

**Figure 9:**
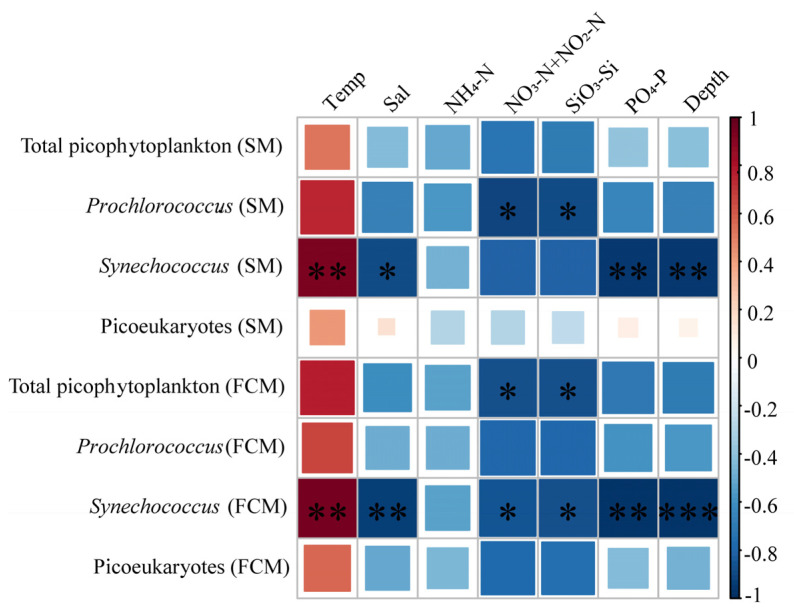
Pearson correlation heat map between the picophytoplankton and environmental factors based on the SM and FCM methods. Red represents positive correlations, and blue represents negative correlations. * indicates a significant correlation between picophytoplankton and environmental factors. *: *p* < 0.05, **: *p* < 0.01, ***: *p* < 0.001.

A Pearson correlation analysis was used to compare the correlations between picophytoplankton and environmental factors according to the SM and FCM methods. The results of both methods showed that temperature was the main environmental factor regulating the vertical distribution of the three picophytoplankton groups in the euphotic zone. Nutrients and salinity were negatively correlated with picophytoplankton in the vertical profile, especially for *Synechococcus* (*p* > 0.05, Figure 9). The only difference was that the picoeukaryotes detected with the SM showed certain positive correlation with salinity, depth, and PO_4_-P, but the correlation was low and insignificant (*p* > 0.05).

## 4. Discussion

In this study, a combination of FCM and SM was used to study the vertical distribution and sizes of picophytoplankton in a euphotic zone (200 m water column) in the Western Pacific Ocean. In terms of vertical distribution patterns, the results of FCM were consistent with those of previous studies in tropical oligotrophic seas [42,60,61], and the results of the SM were consistent with those of FCM. In the absence of a direct measurement of cell size, we estimated the mean cell size by using flow cytometry with forward scattering. Our estimates of the picophytoplankton size were comparable to those reported in previous studies. For example, the diameter of *Prochlorococcus* reported by Chisholm et al. [62] was between 0.6 and 0.8 μm, and that found by Morel et al. [63] was between 0.54 and 0.67 μm. In addition, Sieracki et al. [64] observed the diameter of *Prochlorococcus* in the Sargasso Sea by using charge-coupled device (CCD) cameras and found it to be in the range of 0.45–0.75 μm. As for the diameter of *Synechococcus*, in the Sargasso Sea, DuRand et al. [1] found that it was 0.74–1.22 μm, Blanchot et al. [42] estimated that the diameter was 0.87–0.95 μm by using FCS, and Shalapyonok et al. [11], who used the same method to estimate the average values of the diameter of *Synechococcus* in a surface mixed layer and below the mixed layer, found sizes of 0.91–0.95 and 0.98–1.14 μm, respectively. In this study, we used FCS to estimate the diameters of *Prochlorococcus* and *Synechococcus* within the range reported above. However, our estimate of the picoeukaryotes’ diameter was slightly smaller than previously reported. Blanchot et al. [42] estimated the diameter of picoeukaryotes in the Western Pacific Warm Pool to be between 1.98 and 2.16 μm with FCS. However, the equivalent spherical diameter of picoeukaryotes estimated with other methods was 1–2 μm; for example, Calvo-Díaz et al. [65] estimated that the diameter of picoeukaryotes was between 1.35–2.05 μm by using side scatter (SSC). There may have been a deviation caused by using too few sampling stations. Nevertheless, in general, the results of the evaluation with the SM for the vertical distribution and diameter of picophytoplankton were consistent with those of FCM, which indicated that it is meaningful to further analyze the composition of picophytoplankton by using the SM.

The results of the SM showed that picoeukaryotes had a high diversity, except for *Prochlorococcus* and *Synechococcus*. The picoeukaryotes detected with the SM mainly included Prymnesiophycea (mainly *Chrysochromulina* and *Phaeocystis*), Cryptophyceae (mainly *Hemiselmis*), Chrysophyta (Chrysophyceae spp. and Pelagophyceae spp.), Dinoflagellata (mainly Gymnodiniales spp.), and Mamiellophyceae. Among them, some species of Prymnesiophycea, Chrysophyta, and Mamiellophyceae, which are picoeukaryotic phytoplankton, were confirmed [39,54,56,57,66,67]. However, the SM detected a high abundance of Cryptophyceae and Dinoflagellata among picophytoplankton, though they are generally considered to be nano-phytoplankton (2–20 μm). In addition, a small number of coccolithophore (e.g., *Ochrosphaera* and *Emiliania*) and diatom (*Chaetoceros*) sequences were detected in the picophytoplankton dataset. This may have been due to errors caused by particle filtration, as the filter allowed cells larger than the nominal apertures to pass through, although we adopted pre-filtration and low-pressure filtration. Cryptophyceae typically lack fibrous cell walls, making them vulnerable to fragmentation; thus, their sequences were abundant in the smallest particle size range (0.2–0.6 μm). The genomes of Dinoflagellates are larger than those of other phytoplankton, resulting in a relatively high number of reads [68]. Nevertheless, Gymnodiniales and diatoms smaller than 2 μm have actually been reported [69]. These results suggest that the diversity of pico-dinoflagellates and pico-diatoms should be emphasized. However, the coccolith sequence that was detected may have been from coccolithophores in their early development or from the base plates that they shed [58,70].

Picoeukaryotic phytoplankton were not evenly distributed in the euphotic zone (Figure 6). The adaptation of picoeukaryotes to light and nutrients, as well as their own ecological characteristics, may explain the different vertical distribution patterns of the different picoeukaryote groups [71,72]. *Chrysochromulina*, for example, as a species responsible for algal blooms, usually appears on the surface, where nutrients are depleted, especially when silicate levels are low, which is conducive to the growth of *Chrysochromulina* [73]. In the case of *Pelagomonas*, light and nutrients are important environmental factors regulating its vertical distribution pattern [74]. *Pelagomonas* is more adaptable to low light and high nutrient contents [75], and the small cell size limits the large-scale energy storage in *Pelagomonas* [76]. Therefore, compared with the surface layer, pico-*Pelagomonas* was distributed more at the bottom of the euphotic layer with a lower light intensity and greater amounts of nutrients and salts. The vertical distribution of *Amoebophrya* may be consistent with that of its host.

The Pearson correlation analysis with the environmental factors showed that temperature was the main environmental factor affecting the vertical distribution of picophytoplankton in the oligotrophic euphotic layer, which is consistent with the findings of previous studies (Figure 9) [77]. *Synechococcus*, in particular, showed a significant positive correlation with temperature (*p* < 0.01), suggesting that the growth of *Synechococcus* was more temperature dependent, and also suggesting its demand for light [29,78]. Although it has been proven that low-light-adapted Prochlorococcus spp. can grow in deep euphotic zones, it is still regulated by temperature [77,79]. Previous studies reported that the optimal temperature for *Prochlorococcus* was 24–25 °C [10,78,80], but *Prochlorococcus* cannot use nitrate due to its lack of nitrate reductase, which makes its presence positively correlated with temperature [79,81,82] but negatively correlated with nitrate and other nutrients that increase with increasing depth [10]. The abundance of picoeukaryotes in general increased with temperature because, with increasing temperature, Chl *a* synthesis increased and light capture gradually increased, thus reducing the limitation of photosynthesis [79,83]. The results of the SM showed that the abundance of picoeukaryotes was positively correlated with salinity, depth, and DIP. Although the correlation was low (*p* > 0.05), it seems that the influence of the environment on picoeukaryotes is complex. This may be due to the complex diversity of picoeukaryotes. As the results of the SM show, picoeukaryotes were not evenly distributed in the euphotic zone (Figure 4 and Figure 6), and some picoeukaryotes had two abundance peaks, which were closely related to environmental factors [66]. In addition, different picoeukaryotes have different preferences for nutrients. Prasinophyceae, Trebouxiophyceae, and Chrysophyceae, for example, prefer higher phosphorus concentrations, whereas Prymnesiophyceae and Cryptophyceae prefer the opposite [84]. According to the results of the SM, some picoeukaryotes did have a high abundance at the bottom of the euphotic zone, such as Dinoflagellates, Pelagophyceae, and unknown taxa [85]. In terms of correlation with environmental factors, the differences between the results of FCM and the SM indicated that community composition should be emphasized in ecological studies of picoeukaryotes, which is another advantage of the SM.

## 5. Conclusions

In this study, we introduced a method for studying the size and species composition of picophytoplankton—size-fractionated filtration plus a high-throughput sequencing molecular method (SM). The results showed that the advantages of SM are different from those of FCM; for example, it can be used to identify a large number of picoeukaryotic phytoplankton taxa, and its estimation of particle size is relatively accurate. Although errors caused by separation and filtration according to particle size are inevitable, SM can be used to accurately evaluate the vertical distribution, diameter, and community composition of picophytoplankton in general, and its advantages may be applicable to large-scale spatial studies of picophytoplankton, such as using specific amplifying primers for different research purposes, combining qPCR for quantification, or combining flow cytometry with sorting to ameliorate the errors caused by size-fractionated filtration. In addition, this approach will make it possible to study the response of individual picophytoplankton taxa to environmental factors.

## Figures and Tables

**Table 1:** Relative abundance of picophytoplankton in different particle size ranges.

	0.2–0.6 μm	0.6–1.2 μm	1.2–2 μm
*Prochlorococcus*	59.86%	27.93%	12.21%
*Synechococcus*	31.46%	29.32%	39.22%
PEuks	14.71%	32.41%	52.88%

**Table 2:** Comparison between the SM and FCM for the particle size evaluation of the three picophytoplankton groups.

	*Prochlorococcus*	*Synechococcus*	Picoeukaryotes
FCM	0.60 ± 0.22 μm	0.83 ± 0.10 μm	1.20 ± 0.47 μm
SM	~0.69 μm	~1.02 μm	~1.21 μm

## Data Availability

The data presented in this study are openly available in the NCBI Sequence Read Achieve database (BioProject: PRJNA748570 and PRJNA747866).
